# The Effect of Furnishing on Perceived Spatial Dimensions and Spaciousness of Interior Space

**DOI:** 10.1371/journal.pone.0113267

**Published:** 2014-11-19

**Authors:** Christoph von Castell, Daniel Oberfeld, Heiko Hecht

**Affiliations:** Johannes Gutenberg-Universität Mainz, Mainz, Germany; McMaster University, Canada

## Abstract

Despite the ubiquity of interior space design, there is virtually no scientific research on the influence of furnishing on the perception of interior space. We conducted two experiments in which observers were asked to estimate the spatial dimensions (size of the room dimensions in meters and centimeters) and to judge subjective spaciousness of various rooms. Experiment 1 used true-to-scale model rooms with a square surface area. Furnishing affected both the perceived height and the spaciousness judgments. The furnished room was perceived as higher but less spacious. In Experiment 2, rooms with different square surface areas and constant physical height were presented in virtual reality. Furnishing affected neither the perceived spatial dimensions nor the perceived spaciousness. Possible reasons for this discrepancy, such as the influence of the presentation medium, are discussed. Moreover, our results suggest a compression of perceived height and depth with decreasing surface area of the room.

## Introduction

In sharp contrast to the many efforts and opinions in the domain of interior design, there is little empirical research regarding the factors that determine perceived room size. Some studies have investigated the relation between lightness and perceived room size (see [1]) but next to nothing is known about the perceptual effects that objects in the room may exert on perceived room size. The latter can be judged holistically in terms of spaciousness [2], [3], [4], or more precisely in terms of spatial dimensions, such as perceived distance between opposite walls or between ceiling and floor. Here we present a first systematic exploration of the effect of objects within the room on perceived room size.

In the field of architecture and interior design, it appears to be a forgone conclusion that objects placed within a room do change the observer’s impression of interior space [5]. We define *objects* as planar formations observed from the outside, and *rooms* as planar formations observed from inside [6], following Thiel’s [7] approach of space establishing elements. The latter approach is based upon Gibson’s [8] ecological theory: “Objects may be thought of as three-dimensional forms existing as separate, isolated visual entities in a larger space than that smaller space which they help establish. [In the context of the *larger* space, the object no longer functions as a space-establishing element and consequently becomes a furnishing (F).]” [7]. In fact, in textbooks on interior design a multitude of assumptions about the effects of furniture on the perception of interior space is made. For the relationship between single objects and the walls behind them, Neufert and Kister [9] assume that bright objects in front of dark walls are perceived as light-weighted, whereas dark objects in front of bright walls are perceived as heavy. According to Brown [10], dark and heavy furnishing is improper for small rooms. Moreover, he suggests a low and minimalistic furnishing as it is common for Japanese flats to make a room appear larger. This enlarging effect of low furnishing has even been proposed for spacious rooms like lofts [11]. In addition, the arrangement of large pieces of furniture along walls or in alcoves and leaving as much surface area uncovered as possible, is said to widen small rooms visually, whereas tall pieces of furniture are said to make a room appear crowded [12]. A guidebook for owners of small rooms advises bright colors in combination with a filigree and compact furnishing [13]. Shaw [14] establishes the rule that every room needs at least one large piece of furniture to serve as an optic center. Likewise, Conran [15] addresses the defining and structuring function of furnishing. And Heuser [16] emphasizes the scale-establishing function of furniture. This implies that the familiar width, depth, or height of a piece of furniture within a room can serve as a cue to determine the size of the room dimensions.

Unfortunately, empirical evidence for virtually all of these detailed assumptions is missing. Astonishingly, the gap between architectural expertise and empirical data has persevered since the late sixties of the 20th century [17].

We found only one study dealing with the impact of furnishing on perceived spatial dimensions. Imamoglu [18] investigated the effect of furnishing on perceived room volume using a full size room with a surface area of 9.82 m^2^. He varied furnishing density in three steps: none, normal, and crowded. From his drawings we reconstructed that about 26% of the surface area was covered by furniture in the normal furnishing condition, and 41% in the crowded furnishing condition. Room volume was judged relative to an 11.95 m^2^ standard room with constant furnishing (we reconstructed that for this room 43% of the surface area was covered by furniture). A negative relation between the amount of furnishing and perceived room volume was reported. Apart from this study, some related evidence supports the notion that sparse furnishing is better than none when attempting to make a room look as large as possible. For instance, Luria, Kinney, and Weissman [19] reported that an additional object between the observer and the target object has an enlarging effect on perceived egocentric distance (in a range from 1.22 m to 4.57 m), irrespective of whether the distance was viewed monocularly or binocularly. Judgments were made relative to a standard distance of 0.61 m. Regarding exocentric distances between two objects, Kundt [20] described a phenomenon, which was later termed Oppel-Kundt-Illusion. He used a horizontal line of defined length, which was perpendicular to the observer’s line of sight. The endings of the line were marked by dots. Additionally, one half of the line was interrupted by further dots, while the other half was not interrupted. The interrupted half was perceived to be longer. Taking the findings of Imamoglu [18], Luria et al. [19], and Kundt [20] together, it becomes clear that the perception of distances and the perception of interior space do not necessarily follow the same rules. Whereas one-dimensional distances, regardless of whether they are egocentric or exocentric, are perceived to be larger when space is filled, the volume of interior space is perceived to be smaller with filled space.

To assure a precise classification of results, we distinguish between the perceived spatial dimensions of a room (volume or size of single room dimensions such as width, depth, or height in meters and centimeters) on the one hand, and the more holistic facets of the affective room impression (e.g. spaciousness, structuredness, or friendliness) on the other hand. Note that this differentiation is not made in the architectural guidelines cited above. With respect to the more holistic facets of the affective room impression (e.g. spaciousness, structuredness, or friendliness), we found six studies that investigate the effect of furnishing. Imamoglu reported an inverse U-shaped relation between the amount of furnishing and spaciousness for full size [18] and model [21] rooms. The rooms with normal furnishing were perceived as most spacious. According to this result, a realtor would do well to furnish a vacant apartment at least sparingly in order to make it look more spacious. For a full size room with constant furniture density, Imamoglu [22] found that perceived spaciousness increases with increasing degrees of organization. Note, however, that for the assessment of spaciousness in the latter three studies, Imamoglu used the Spaciousness-Crampedness-Scale (cf. [21]) which operationalizes spaciousness as a three-dimensional construct (“appeal”, “planning”, and “space freedom”) that is not restricted solely to spaciousness, but seems to reflect a more general affective appraisal of indoor quality. Kaye and Murray [23] used perspective drawings of a living room and varied arrangement as well as density of furnishing. Density had an influence on spaciousness, whereas arrangement did not affect spaciousness. Moreover, arrangement and density had an effect on perceived structuredness. Unfortunately, the directions of the effects were not reported. Using perspective drawings, Wools [24] found an effect of arrangement of furnishing on judgments of the friendliness of the room. Rooms with a casual arrangement of easy chairs and a cocktail table were judged to be friendlier compared to rooms with a strict chairs and desk arrangement. A cross-cultural study by Mak and Ng [25] shows high correspondence between furnishing recommendations for the same room provided independently by western (Sydney) and eastern (Hong Kong) architects. Both conformed to Feng-Shui rules.

Taken together, expert opinion and experimental findings suggest that furnishing does influence both the perception of the spatial dimensions as well as perceived spaciousness and other affective judgments. However, little is known about the origin (e.g. early stages of visual perception vs. higher cognitive processes) and the direction of such effects. Two studies by Imamoglu [18], [21] should be highlighted as they provide the working hypotheses for the present study: (a) a room with medium furnishing should look more spacious compared to no furnishing or over-furnishing [18], [21], and (b) a room’s volume should be perceived as larger when unfurnished compared to any degree of furnishing [18].

The investigation of how furniture impacts perceived spatial dimensions and spaciousness is fraught with a multitude of potential confounding variables, such as roughness and permeability (e.g. window front vs. concrete wall) of walls, surface area, lighting conditions, and room proportions. Stamps and Krishnan [26] and Stamps [27], [28], [29] listed these factors in the context of perceived spaciousness. With respect to perceived room volume as dependent variable, several studies show effects of room proportions. Holmberg, Küller, and Tidblom [30], Holmberg, Almgren, Söderpalm, and Küller [31] and Sadalla and Oxley [32] reported that the volume of rectangular model rooms and full size rooms with constant surface area and height was perceived larger with increasing depth, relative to a square room of constant volume. Regarding the verbal estimation of one single room dimension on a centimeter scale, Oberfeld, Hecht, and Gamer [33] and Oberfeld and Hecht [1] found effects of ceiling lightness and wall lightness on perceived room height. Perceived room height increased with ceiling lightness as well as with wall lightness.

Keeping this multitude of potential confounding variables in mind, laboratory experiments with full size standardized rooms seem prohibitive. We have considered two alternatives, (a) true-to-scale model rooms that preserve all physical characteristics of real size rooms, on a scale of 1∶10, for example, or (b) three-dimensional simulations in virtual reality (VR). Both alternatives allow for an efficient manipulation of independent variables and a sufficient control of potential confounding variables. Following this consideration, we used true-to-scale model rooms in Experiment 1 and virtual rooms in Experiment 2.

## Experiment 1: Variation of Furnishing in Model Rooms

Experiment 1 was conducted to explore effects of furnishing on perceived spatial dimensions and spaciousness of true-to-scale model rooms. According to the current state of research and the advice provided by architects and designers, we hypothesized that furnishing influences both the perceived spatial dimensions as well as the perceived spaciousness of the model rooms. Furnishing should cause a decrease in perceived spatial dimensions, but perceived spaciousness should increase with furnishing.

### Method

#### Ethics statement

In accordance with the Declaration of Helsinki, all participants gave their informed written consent, after the topic and potential risks of the study had been explained to them. After the experiment, participants were debriefed about the intention of the experiment. Prior to the study, the Institutional Review Board of the Department of Psychology at the Johannes Gutenberg-Universität informed us that in accordance with the department’s ethics guidelines no explicit ethics vote of the IRB was necessary for our study, because only harmless visual stimuli were presented, no physiological parameters were measured, and no misleading or wrong information was given to participants.

#### Participants

A total of 80 observers (58 women and 22 men), aged from 18 to 64 (*M* = 24.87 years, *SD* = 7.28 years), with normal or corrected to normal vision participated voluntarily in Experiment 1. All participants came from a region that uses the metric system and were uniformed about the objective of the experiment.

#### Apparatus

**Figure 1:**
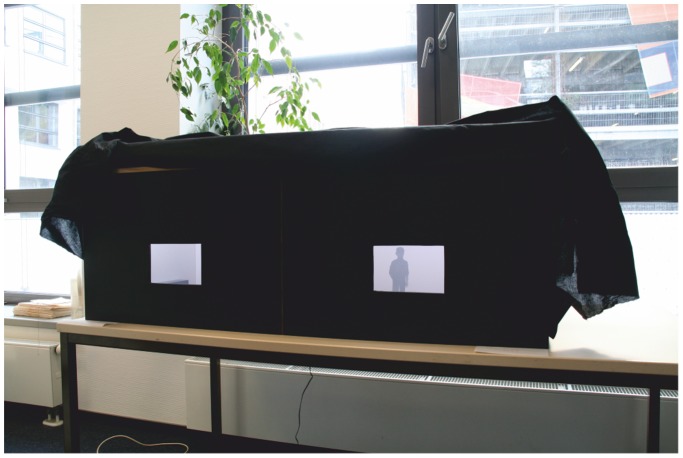
Experiment 1: Exterior view of the experimental setup.

Two identical 1∶10 scale wooden models of a square room with 16 m^2^ surface area and 2.70 m ceiling height were constructed for this experiment. Front walls were replaced by a black (RAL 9005) wooden cover that contained a viewport for each room. The latter consisted of a rectangular opening (16 cm wide and 12.50 cm high), horizontally centered 9 cm above the floor of the room (see Figure 1). Subjects were asked to place their forehead against the top edge of the viewport. Eye height was 16 cm above the floor, corresponding to 1.60 m at full scale. The interior surfaces were painted white (walls and ceilings, RAL 9010) and grey (floors, RAL 7005). Rooms were lighted by 18 LEDs mounted above the viewport and invisible to the subject. Luminance intensity was 140 lux in the middle of the floor. Additionally, the experimental setup was covered by a black tarp to prevent external light entering through the viewports (see Figure 1).

**Figure 2:**
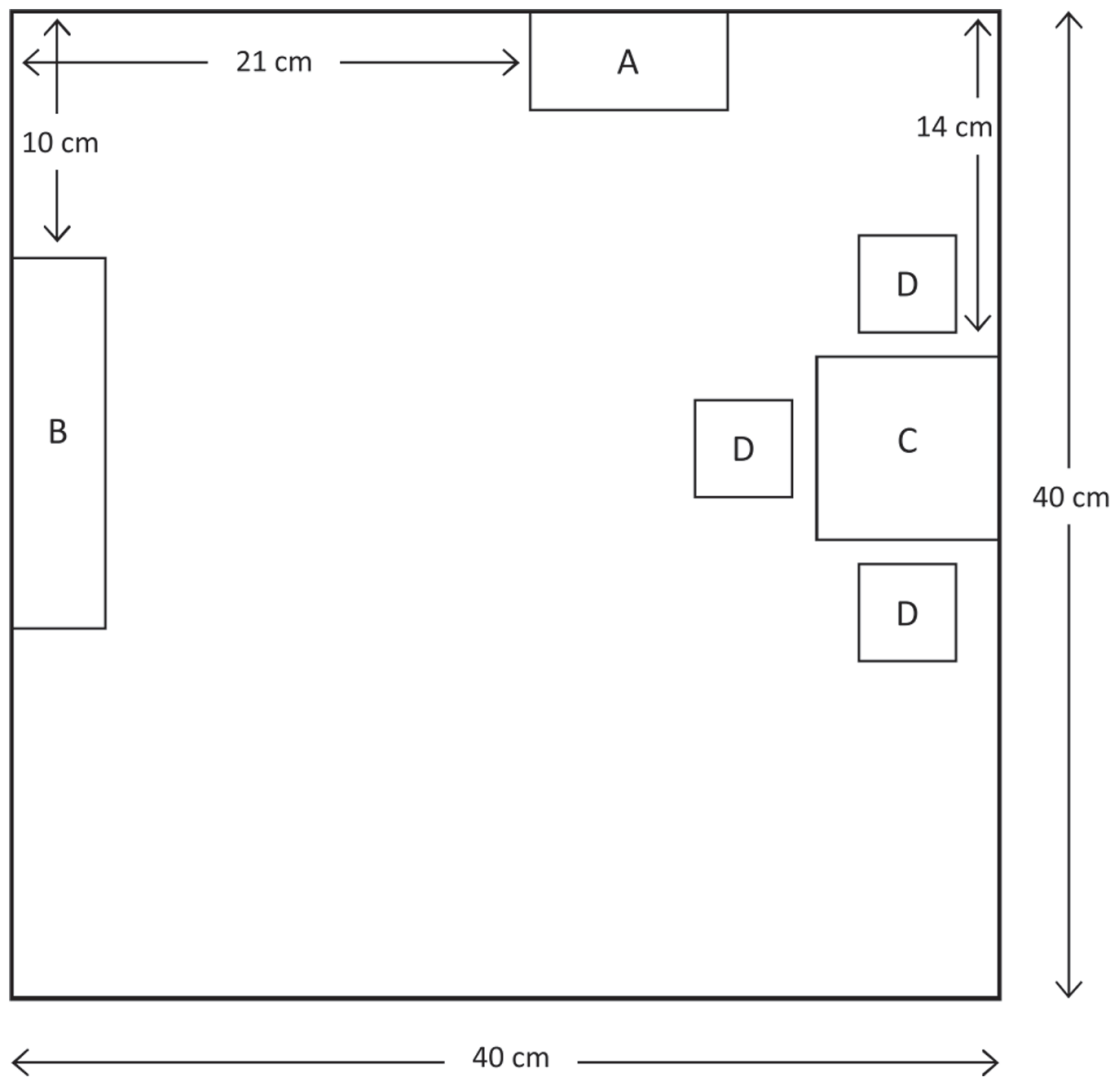
Experiment 1: Bird’s eye view of the arrangement of cuboids in the furnished model room. Objects D were with respect to C at a distance of 1 cm. The bottom horizontal line corresponds to the front wall.

**Figure 3:**
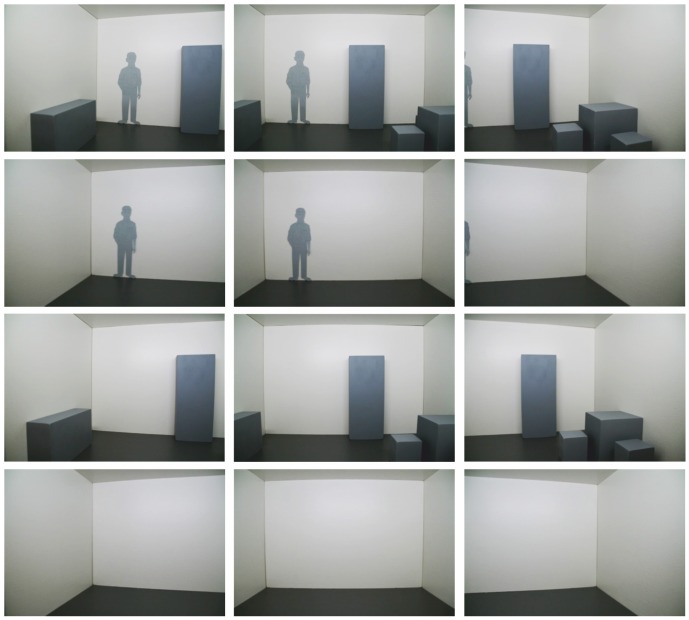
Experiment 1: Interior view in the maximum left, default, and maximum right viewing direction of the furnished (first row) and the unfurnished (second row) model room with the additional scaling cue as well as of the furnished (third row) and the unfurnished (fourth row) model room without the additional scaling cue.

The two rooms were positioned side by side on a table. In the room on the left-hand side, light grey cuboids (RAL 9001) representing abstract pieces of furniture were installed. A “cupboard” (A; width×depth×height = 8×4×19 cm), a “sideboard” (B; 15×3.80×7.40 cm), a “table” (C; 7.40×7.40×7.40 cm) and three “stools” (D; 3.90×3.90×4.50 cm) fitting the specifications of a large Swedish furniture company were arranged as shown in Figure 2. This arrangement was chosen in accordance with the architectural assumption that small rooms with furniture arrangements along walls and a high percentage of uncovered surface area are perceived as being larger [12]. Furthermore, the chosen arrangement allowed for optimal visibility through the viewport (see Figure 3).

An additional scaling cue was presented to half of the observers: A 17.80 cm tall silhouette of a male person was fixed on the rear wall of the rooms to scale the rooms’ physical size. Note that 1.78 m is approximately the average body height of male Germans [34]. No scaling cue was presented to the other half of the observers (see Figure 3).

#### Design and procedure

Four factors were varied in Experiment 1.

1. Furnishing was varied as within-subjects factor. The furnished and the unfurnished room were presented to all participants.
2. As binocular vision is known to provide important cues about the spatial layout within a range up to two meters around the observer’s eyes, referred to as personal space [35], one group of observers (*n_1_* = 40) viewed the rooms monocularly, while the second group (*n_2_* = 40) viewed them binocularly.
3. The presence or absence of a scaling cue was varied as a between-subjects factor. One half of the subjects in each viewing condition judged the rooms with the scaling cue, the other half judged the rooms without scaling cue.
4. To control for order effects, the presentation order was additionally varied as a between-subjects factor. One half of the subjects at each factor level combination of viewing condition and scaling cue judged the furnished room first, the other half judged the unfurnished room first.

Observers looked through the viewport and estimated either width, depth, and height of the corresponding full scale room (with scaling cue), or width, depth, and height of the model room (without scaling cue) as well as spaciousness. Subjects noted their estimates regarding the size of the room dimensions on a questionnaire. Estimates were noted in meters and centimeters (with scaling cue) or in centimeters (without scaling cue). Consistent with various previous studies (e.g., [2], [3], [4], [26], [27], [28], [29], [36], [37], [38]), perceived spaciousness was judged on a rating scale with 10 ordered response categories between a contrastive pair, ranging from “narrow” (value 0) to “spacious” (value 9) [German: “eng” to “weit”]. The comparatively high number of response categories was chosen because reliability of rating scales increases with higher differentiation (e.g., [39], [40], [41]) while validity scores do not decrease (e.g. [41]). No time limit was given. The experiment was conducted in a quiet corner of a library at the University of Mainz. Subjects were instructed in written form. Further inquiries were answered by the experimenter. Subjects were tested individually in single sessions. The experiment lasted approximately 5 to 10 minutes.

In the monocular viewing condition, the rooms were presented to the observer’s dominant eye. Before the experiment, subjects were asked to fixate a black dot at a distance of 4 m through a pipe (25 cm long, 2.5 cm diameter) with an eye of their choice. The chosen eye was defined as dominant. The non-dominant eye was covered by an eye patch.

### Results and Discussion

In summary, with furniture, the rooms were rated to look higher, but less spacious, as compared to the no-furniture condition. We will now first describe our *analysis methods*, then the dependent variable of *perceived spatial dimensions*, and then *perceived spaciousness*.

#### Analysis methods

**Figure 4:**
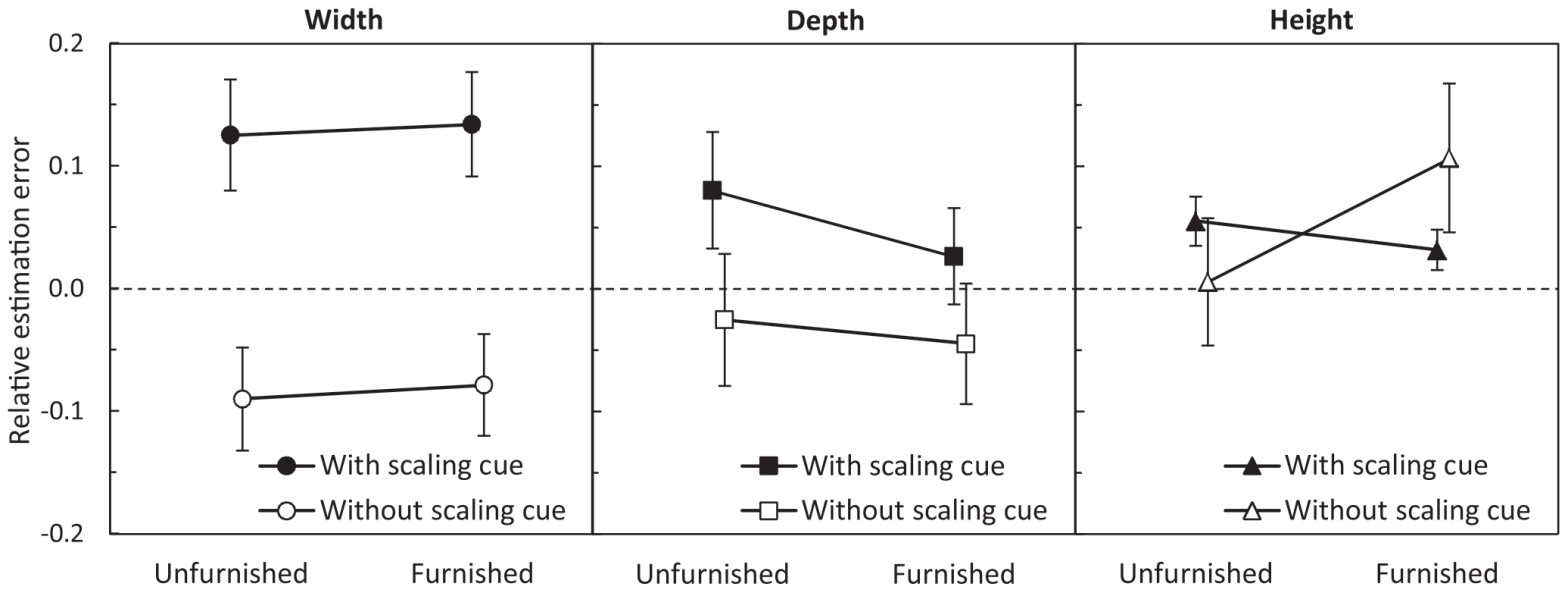
Experiment 1: Mean relative estimation errors of the three room dimensions as a function of furnishing and scaling cue. Error bars show ±1 SEM of the 39 individuals in the factor level combinations with scaling cue and of the 40 individuals in the factor level combinations without scaling cue.

Regarding perceived spatial dimensions, data were analyzed in terms of the relative estimation error (see Figure 4). This is defined by EstError_rel_ = (size_est_/size_phys_) − 1, where EstError_rel_ is the relative estimation error on the respective room dimension, size_est_ is the estimated size of the respective room dimension and size_phys_ is the physical size. Values above zero imply overestimation, values below zero imply underestimation. The relative estimation errors of the three room dimensions were calculated separately for both rooms.

We conducted a multivariate outlier analysis for the relative estimation errors. For this purpose, we averaged the three relative estimation errors over both rooms and calculated Mahalanobis distances [42] indicating the multivariate difference between one subject’s estimate and the group’s mean estimate. These distances are defined by Δ^2^ *_ij_* = (*x_i_* − µ*_j_*)*^t^* Σ*_j_* ^−1^ (*x_i_* − µ*_j_*), where Δ^2^ *_ij_* is the squared Mahalanobis distance of subject *i* in group *j*, (*x_i_* − µ*_j_*)*^t^* is the transposed difference vector between the relative estimation error vector *x_i_* for subject *i* and the group mean vector µ*_j_* of group *j* on the three room dimensions, and Σ*_j_* ^−1^ is the inverse of the variance covariance matrix of the relative estimation errors of all subjects in group *j* on the three room dimensions. Group means and variance covariance matrices were calculated separately for the judgments in the monocular and the binocular condition as well as for the judgments with and without scaling cue. For every subject, one Mahalanobis distance was calculated. The squared Mahalanobis distances were tested against a critical value defined by Penny [43]. One subject from the factor level combination “binocular viewing condition with scaling cue” exceeded the critical value, which is 10.49 for *n* = 20 subjects and *p* = 3 dependent variables. This subject was excluded from further analyses regarding the perceived spatial dimensions.

#### Perceived spatial dimensions

A doubly multivariate repeated-measures analysis of variance (MANOVA) was conducted. Furnishing was a within-subjects factor, viewing condition, scaling cue, and presentation order were between-subjects factors, and the three relative estimation errors were the dependent variables. As a post-hoc analysis, repeated-measures ANOVAs were conducted separately for each room dimension (cf. Table 1). To control the familywise Type I error rate, we tested against Bonferroni-Holm adjusted alpha values [44]. This correction was also applied to all further multiple comparisons.

**Table 1:** Experiment 1: Results of the MANOVA and the post-hoc calculated repeated measures ANOVAs conducted for the relative estimation errors of the room dimension estimates.

Source	*V*	*F*	*df_source_*	*df_error_*	*p*	α_corr_	partial η^2^
**Furnishing (F)**	**.121**	**3.175**	**3**	**69**	.029*		**.121**
Width		0.233	1	71	.631	.050	.003
Depth		2.999	1	71	.088	.025	.041
Height		4.261	1	71	.043	.017	.057
**Viewing condition (VC)**	**.092**	**2.326**	**3**	**69**	**.082**		**.092**
**Scaling cue (SC)**	**.271**	**8.563**	**3**	**69**	<.001*		**.271**
Width		14.811	1	71	<.001*	.017	.173
Depth		2.164	1	71	.146	.025	.030
Height		0.049	1	71	.825	.050	.001
**Presentation order (PO)**	**.043**	**1.039**	**3**	**69**	**.381**		**.043**
**F×SC**	**.138**	**3.674**	**3**	**69**	.016*		**.138**
Width		.004	1	71	.952	.050	.000
Depth		.679	1	71	.413	.025	.009
Height		11.199	1	71	.001*	.017	.136

*Note: V* indicates the multivariate test statistic Pillai’s trace, *α_corr_* indicates the Bonferroni-Holm adjusted alpha level. Partial η^2^ is reported as a measure of effect size. All other effects were not significant (all *p*-values >.05). Significant *p*-values are marked by an asterisk. Multivariate effects are printed in bold font.

The MANOVA showed a significant main effect of furnishing. As seen in Figure 4, perceived depth slightly decreased with furnishing, whereas perceived height slightly increased with furnishing. The mean width estimates were virtually not affected by furnishing. According to the post-hoc repeated-measures ANOVAs, this effect cannot be attributed to the perceived spatial extension of one single room dimension. However, without the correction for multiple testing, the effect of furnishing on the perceived height was significant and, in tendency, there was an effect of furnishing on the perceived depth. As the MANOVA takes also the correlations between the dependent variables into account, this result is not uncommon. There was a significant furnishing×scaling cue interaction. The post-hoc univariate rmANOVAs showed that this effect can be traced back to the perceived height: Furnishing only increased perceived height when the scaling cue was absent (see Figure 4). In other words, the scaling cue moderated the relation between furnishing and perceived height. As a measure of effect size, we calculated Cohen’s *d_z_* [45] for the influence of furnishing on perceived height without the scaling cue. This is defined by *d_z_* = *M_D_*/*SD_D_*, where *M_D_* is the mean of the differences between the height estimates in the furnished and the unfurnished condition without the scaling cue and *SD_D_* is the standard deviation of the differences. The effect size was *d_z_* = 0.49, indicating a weak to medium effect of furnishing on perceived height when no object of familiar height was present.

The multivariate main effect of the scaling cue was significant (cf. Table 1). As seen in Figure 4, the observers judged the rooms to be wider and deeper when the scaling cue was present as compared to absent. In the post-hoc univariate rmANOVAs, the effect of scaling cue was only significant for the width estimates. According to Ittelson [46] and Epstein, Park, and Casey [47], the familiar size of objects (e.g. coins, playing cards) serves as an important monocular cue for the perception of egocentric distances. The familiar size of the silhouette appears to have also served as a monocular cue for the observers’ estimates of width, being an exocentric distance from the observer’s point of view. Interestingly, in the MANOVA the main effect of viewing condition was not significant. This indicates either that observers based their estimates mainly on monocular cues such as height in the visual field, familiar size of objects, and linear perspective, or binocular vision provided no additional information for the perception of the spatial layout of the model rooms. According to the MANOVA, all other effects were not significant (all *p*-values >.05).

Were observers really judging the size of single room dimensions or rather the overall room volume? To answer this question, we analyzed the pattern of the relative estimation errors of width, depth, and height (see Figure 4) in greater detail. Provided that our subjects had to judge the size of single room dimensions, our results should be compatible with prior findings on the perceived spatial extent of single room dimensions in three-dimensional layouts. When no scaling cue was present, the pattern was compatible with prior findings on the horizontal-vertical illusion [48], according to which exocentric distances in the vertical plane are estimated larger than in the horizontal plane. According to Higashiyama [49], this optical illusion also influences the perceived size of exocentric distances on building walls observed from the outside. To test whether there was a differential compression or extension of the spatial dimensions in our data, we conducted *t*-tests for paired samples for width vs. depth, height vs. width, and height vs. depth, separately for both levels of scaling cue as well as for both levels of furnishing. As shown in Table 2, there was a consistent overestimation of height compared to width when the scaling cue was absent, irrespective of furnishing. Furthermore, for the conditions without scaling cue, there was no significant difference between the relative estimation errors of width and depth, indicating that observers perceived square surface areas in both rooms. Taken together, our data provide some evidence for the assumption that observers really judged the size of the room dimensions when the scaling cue was absent.

**Table 2:** Experiment 1: Results of the paired t-tests (test value = 0) conducted for the difference of the relative estimation errors in the comparisons width vs. depth, height vs. width, and height vs. depth, differentiated according to scaling cue and furnishing.

Scaling cue	Furnishing	Comparison	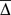	*SE* 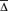	*t*	*df*	*p*	α_corr_
Yes	Yes	Width vs. depth	0.108	0.041	2.596	38	.013*	.025
		Height vs. width	−0.103	0.039	−2.630	38	.012*	.017
		Height vs. depth	0.006	0.038	0.132	38	.896	.050
	No	Width vs. depth	0.045	0.051	0.874	38	.388	.025
		Height vs. width	−0.070	0.040	−1.731	38	.092	.017
		Height vs. depth	−0.025	0.048	−0.516	38	.609	.050
No	Yes	Width vs. depth	−0.034	0.030	−1.138	39	.262	.050
		Height vs. width	0.185	0.042	4.382	39	<.001*	.025
		Height vs. depth	0.151	0.033	4.554	39	<.001*	.017
	No	Width vs. depth	−0.064	0.035	−1.866	39	0.070	.025
		Height vs. width	0.100	0.027	3.594	39	0.001*	.017
		Height vs. depth	0.031	0.033	0.939	39	0.354	.050

*Note:* α*_corr_* indicates the Bonferroni-Holm adjusted alpha level. Significant *p*-values (two-tailed) are marked by an asterisk.

#### Perceived spatiousness

**Figure 5:**
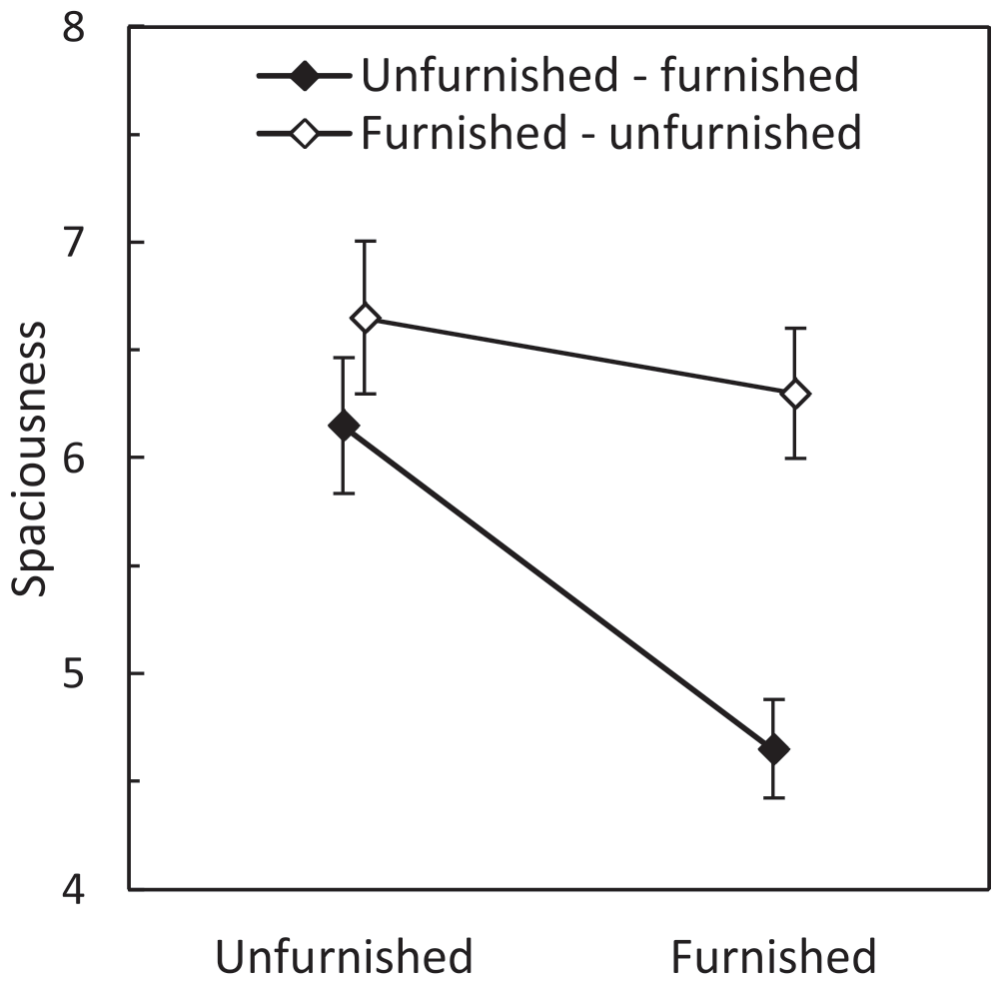
Experiment 1: Mean spaciousness judgments as a function of furnishing and presentation order. Error bars show ±1 SEM of the 40 subjects in each factor level combination.

Figure 5 displays the average ratings of spaciousness**.** The furnished room was perceived as less spacious, and both rooms were perceived as more spacious when the furnished room was presented first. Furthermore, the difference in perceived spaciousness between the unfurnished and the furnished room was larger when the unfurnished room was presented first.

A repeated-measures ANOVA with furnishing as within-subjects factor, viewing condition, scaling cue, and presentation order as between-subjects factors, and perceived spaciousness as dependent variable was conducted. The main effect of furnishing was significant, *F*(1, 72) = 12.068, *p* = .001, partial η^2^ = .144. Rather surprisingly, furnished model rooms were judged to be less spacious than unfurnished rooms. The effect size for the mean of the differences between the spaciousness judgments in the unfurnished and the furnished condition was *d_z_* = 0.38 [45], indicating a weak effect of furnishing on perceived spaciousness. The main effect of presentation order was also significant, *F*(1, 72) = 11.124, *p* = .001, partial η^2^ = .134. Furthermore, the interaction effect of furnishing and presentation order was significant, *F*(1, 72) = 4.663, *p* = .034, partial η^2^ = .061. As a side effect, the ANOVA showed a significant three-way interaction of viewing condition, presentation order, and scaling cue, *F*(1, 72) = 6.551, *p* = .013, partial η^2^ = .083. All other effects were not significant (all *p*-values >.05).

Taken together, we found a weak effect of furnishing on perceived spaciousness. Furnished model rooms looked less spacious. The main effect of presentation order is compatible with the architectural assumption that, besides “intrinsic” physical room characteristics, even “extrinsic” factors, such as a room seen before, influence the observer’s room impression [6]. Note that unlike perceived spatial dimensions, perceived spaciousness was not influenced by the scaling cue. This underlines the holistic character of spaciousness, which is not easily affected by a single cue for the size of single room dimensions.

## Experiment 2: Independent Variation of Furnishing and Surface Area in Vr

Experiment 2 was conducted to explore the effects of furnishing and surface area on perceived spatial dimensions and spaciousness of three-dimensional full scale room simulations in VR. Thus, this experiment was both aimed at replicating and specifying the findings of Experiment 1 as well as at comparing the two methodological pathways mentioned above – true-to-scale model rooms and VR. With reference to the results of Experiment 1, we expected that, other things being equal, furnishing would increase perceived height and decrease perceived spaciousness of the room simulations in VR. To test whether this effect of furnishing can be generalized across different surface areas, physical width and depth were varied additionally. As furnishing is said to interact with the characteristics of the surrounding room, for example, in particular furnishing recommendations for small rooms (see Introduction), we expected that there is an additional furnishing×surface area interaction on perceived height and spaciousness.

### Method

#### Ethics statement

In accordance with the Declaration of Helsinki, all participants gave their informed written consent, after the topic and potential risks of the study had been explained to them. After the experiment, participants were debriefed about the intention of the experiment. Prior to the study, the Institutional Review Board of the Department of Psychology at the Johannes Gutenberg-Universität informed us that in accordance with the department’s ethics guidelines no explicit ethics vote of the IRB was necessary for our study, because only harmless visual stimuli were presented, no physiological parameters were measured, and no misleading or wrong information was given to participants.

#### Participants

A total of 40 observers (29 women and 11 men), all of whom well acquainted with the metric system, aged from 20 to 41 (*M* = 25.53 years, *SD* = 4.27 years) took part voluntarily in Experiment 2. All participants were uniformed about the hypotheses of the experiment. None of the subjects had participated in Experiment 1. All subjects reported normal or corrected-to-normal vision, and their visual acuity was tested before the experiment. For that purpose, we used Landolt rings to test visual acuity and a digital version of the Titmus-test [50] with stereoscopic disparities of 800, 400, 200, 140, 100, 80, 60, 50, and 40 seconds of arc to test stereoscopic vision. In the latter test, the criterion for participation in the experiment was that at least 6 of the 9 trials were answered correctly.

#### Apparatus

We used seven virtual rooms with a square surface area and a constant height of 2.70 m. Width and depth were varied between 4 m and 10 m in steps of 1 m. Thus, the surface area was varied between 16 m^2^ and 100 m^2^. Note that, in terms of spatial proportions, the smallest room corresponded exactly to the model rooms used in Experiment 1. Rooms were presented both unfurnished and furnished. Furnished rooms were equipped with full size simulations of the cuboids used in Experiment 1. The amount and arrangement of furnishing was kept constant in all seven rooms causing the density of furnishing to decrease with increasing physical room size. The percentage of surface area covered by furniture varied between 11.84% for the smallest and 1.89% for the largest room. A constant arrangement of furnishing was realized by positioning the furniture objects directly against the walls, and fixing their position along the wall (see Figure 2). For example, the center of the cupboard divided the room width from the left to the right at a constant ratio of 5∶3.

**Figure 6:**
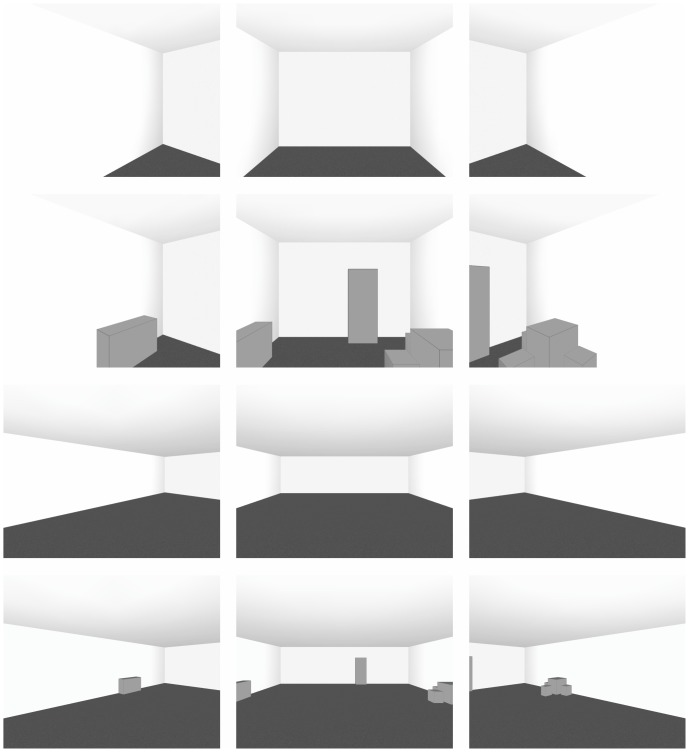
Experiment 2: Two-dimensional screenshots of the smallest (above) and the largest (below) virtual room in the furnished and unfurnished condition, each displayed from the leftmost, the default, and the rightmost vantage point.

Walls and ceiling were overlaid with a white fine-grained texture. The floor was overlaid with a medium grey fine-grained texture. Shadows at the room edges were provided for a more realistic lighting impression. Cuboids were colored in light grey. Thin dark grey lines accentuated the cuboids’ edges. Luminance intensity was kept constant in all conditions. Figure 6 shows screenshots of the smallest and the largest virtual room.

The virtual rooms were displayed using Vizard [51] on a Pentium IV computer with a NVIDIA QuadroFX3500 graphics board and presented on a 2.60 m wide and 1.95 m high screen (aspect ratio 4∶3). The stereoscopic projection was generated by a 3D rear-projector (projectiondesign F10 AS3D) with a resolution of 1,400×1,050 pixels and a color depth of 32 bits. Subjects wore LCD shutter glasses (XPAND X102) whose shifting time was synchronized with the projector’s refresh rate via an infrared connection. The projector’s refresh rate was 120 Hz, providing each eye with 60 pictures per second. The individual inter-pupillary distance of each subject was measured before the experiment and taken into account in computing the stereoscopic disparity of the two images.

During the experiment, subjects sat on a height-adjustable chair. A chin rest provided for a constant eye position which was horizontally and vertically centered to the projection screen. The distance between the observer’s eyes and the projection screen was 2 m. The enclosed visual angle was 66° horizontally and 52° vertically.

**Figure 7:**
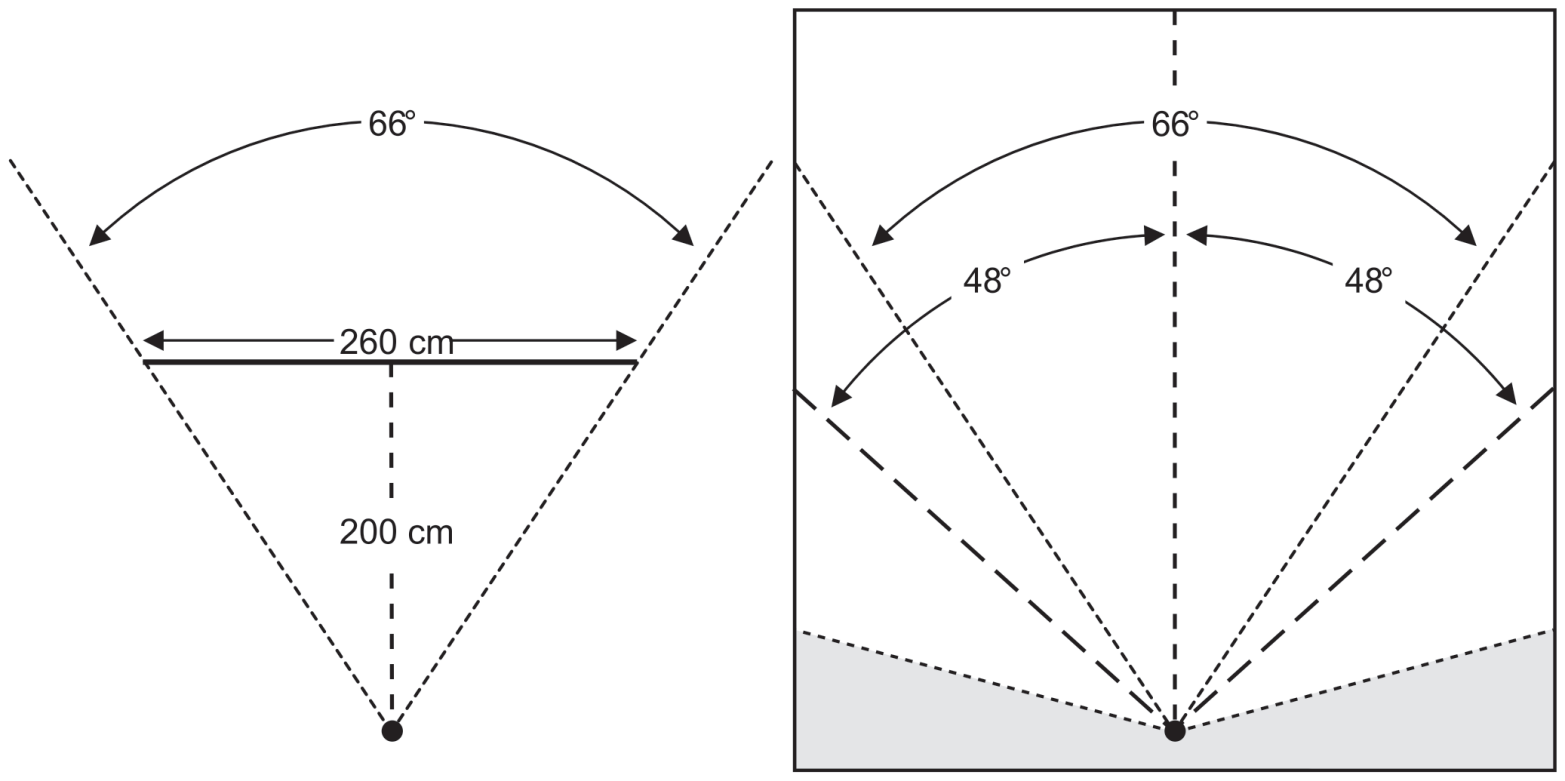
Experiment 2: The observer’s position relative to the projection screen (left-hand side) and relative to the smallest virtual room (right-hand side). Within the virtual room, the grey shaded area was not visible to observers.

The observer’s virtual position was 20 cm in front of the virtual room’s (invisible) front wall, horizontally centered inside the virtual room. Virtual eye height was set at 1.60 m. Subjects were told that their virtual position was like leaning with their back against the horizontal center of the virtual room’s front wall. The default setting of the virtual viewing direction was horizontally and vertically perpendicular to the virtual room’s rear wall. Using a mouse wheel, subjects could turn the virtual horizontal direction of the 66°-wide viewing angle 48° to the left and 48° to the right. Thus, the total visible horizontal viewing angle was 162° (see Figure 7).

#### Design and procedure

Two factors were varied in a fully crossed repeated measures design.

1. Furnishing was varied on two levels (furnished, unfurnished).
2. The surface area was varied on seven levels (16 m^2^, 25 m^2^, 36 m^2^, 49 m^2^, 64 m^2^, 81 m^2^, 100 m^2^), corresponding to depth and width varying from 4 m to 10 m in steps of 1 m.

The resulting 14 factor level combinations were presented in random order. After a period of free exploration of the virtual room, the observer told the experimenter his or her estimate of width, depth, and height in meters and centimeters as well as ratings of spaciousness on the same rating scale as in Experiment 1. For this purpose, the experimenter successively faded in questions about the room’s width, depth, height, and spaciousness in this fixed order in the upper fourth of the screen. The estimates were noted by the experimenter visibly to observers on the screen. While the questions were presented on the screen, the observers could still explore the virtual room. No time limit was given. The experiment was conducted in a dimly lit rectangular room with 105 m^2^ surface area and 2.90 m room height. Subjects were instructed in written form. Further inquiries were answered by the experimenter. Subjects were tested individually in single sessions. The experiment lasted approximately 25 minutes.

### Results and Discussion

In summary, neither did furniture have an effect on the spaciousness judgments, nor did it influence perceived room height. However, a trend to increase both perceived room width and depth could be discerned. First, we describe our *analysis methods*, then the dependent variable of *perceived spatial dimensions*, and then *perceived spaciousness*.

#### Analysis methods

**Figure 8:**
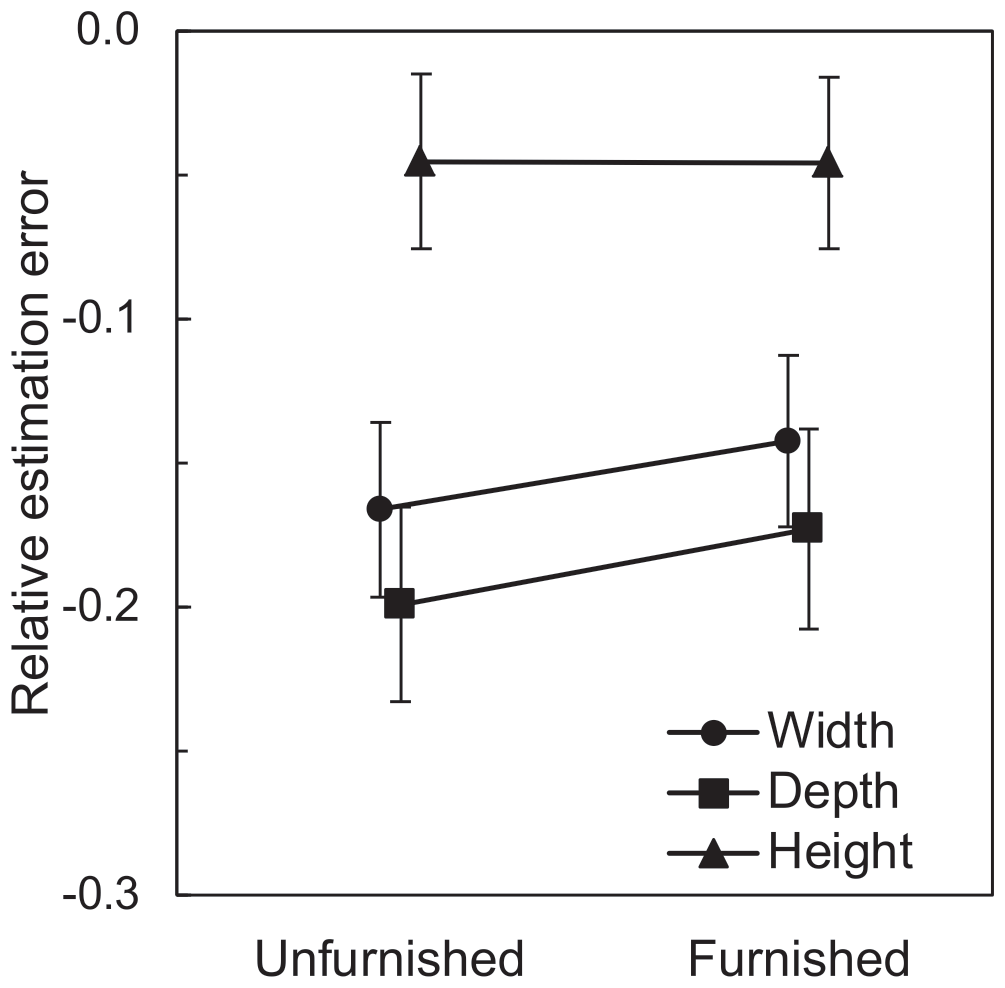
Experiment 2: Mean relative estimation errors of the three room dimensions as a function of furnishing. Error bars show ±1 SEM of the 39 subjects in each condition.

Analogous to Experiment 1, perceived spatial dimensions were analyzed in terms of the relative estimation error. Relative estimation errors were calculated separately for the three room dimensions of the 14 rooms (see Figure 8).

We conducted a multivariate outlier analysis for the relative estimation errors. For this purpose, we averaged the relative estimation errors of width, depth, and height over the 14 rooms and calculated Mahalanobis distances. Due to the fully crossed repeated measures design, the group mean and variance covariance matrix were calculated over all 40 subjects. For every subject, one Mahalanobis distance was calculated. Squared Mahalanobis distances were tested against a critical value defined by Penny [43]. One subject exceeded the critical value of 10.49 for *n* = 20 subjects and *p* = 3 dependent variables (the next higher critical value of 14.18 is defined for *n* = 50 subjects). This subject was excluded from further analyses regarding the estimation of the rooms’ spatial dimensions.

#### Perceived spatial dimensions

A doubly multivariate repeated-measures analysis of variance (MANOVA) was conducted. Furnishing and surface area were within-subjects factors, and the three relative estimation errors were the dependent variables. Figure 8 shows the mean relative estimation errors for the three room dimensions as a function of furnishing. There was a tendency towards higher estimates of width and depth when furniture was present compared to absent. Different from the results of Experiment 1, the perceived room height remained almost constant in both furnishing conditions. The MANOVA showed that the effect of furnishing was not significant (cf. Table 3). An explanation attempt for this discrepancy is provided in the General Discussion.

**Table 3:** Experiment 2: Results of the MANOVA and the post hoc calculated repeated-measures ANOVAs conducted for the relative estimation error of the room dimension estimates.

Source	*V*	*F*	*df*_source_	*df*_error_	*p*	α_corr_	partial η^2^	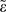
**Furnishing (F)**	**.123**	**1.684**	**3**	**36**	**.188**		**.123**	
**Surface area (SA)**	**.785**	**4.265**	**18**	**21**	.001*		**.785**	
Width		1.809	6	228	.155	.050	.045	.460
Depth		8.084	6	228	<.001*	.025	.175	.441
Height		10.623	6	228	<.001*	.017	.218	.874
**F×SA**	**.580**	**1.614**	**18**	**21**	**.146**		**.580**	

*Note: V* indicates the multivariate test statistic Pillai’s trace, α*_corr_* indicates the Bonferroni-Holm adjusted alpha level. Partial η^2^ is reported as a measure of effect size. 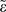 is the correction factor for the degrees of freedom according to Huynh and Feldt [60]. Significant *p*-values are marked by an asterisk. Multivariate effects are printed in bold font.

On average, the virtual room’s spatial dimensions were perceived as compressed, evident in the negative relative estimation errors for all room dimensions (see Figure 8). A Hotelling’s *T^2^*-test (test value = 0) showed a general underestimation of the virtual rooms’ spatial dimensions. In the post-hoc calculated *t*-tests for single samples, the underestimation was significant for width and depth (cf. Table 4).

**Table 4:** Experiment 2: Results of Hotellings T^2^-test [65] and the post-hoc calculated t-tests for single samples (test value = 0) conducted for the means of the relative estimation error of the room dimension estimates.

Room dimension	*T^2^*	*F*	*t*	*df*	*df_source_*	*df_error_*	*p*	α_corr_
**Combined**	**34.127**	**10.777**			**3**	**36**	<.001*	
Width			−5.525	38			<.001*	.025
Depth			−5.556	38			<.001*	.017
Height			−1.818	38			.077	.050

*Note: T^2^* indicates the multivariate test statistic Hotellings *T^2^*, α*_corr_* indicates the Bonferroni-Holm adjusted alpha level. Significant *p*-values (two-tailed) are marked by an asterisk. Multivariate effects are printed in bold font.

**Figure 9:**
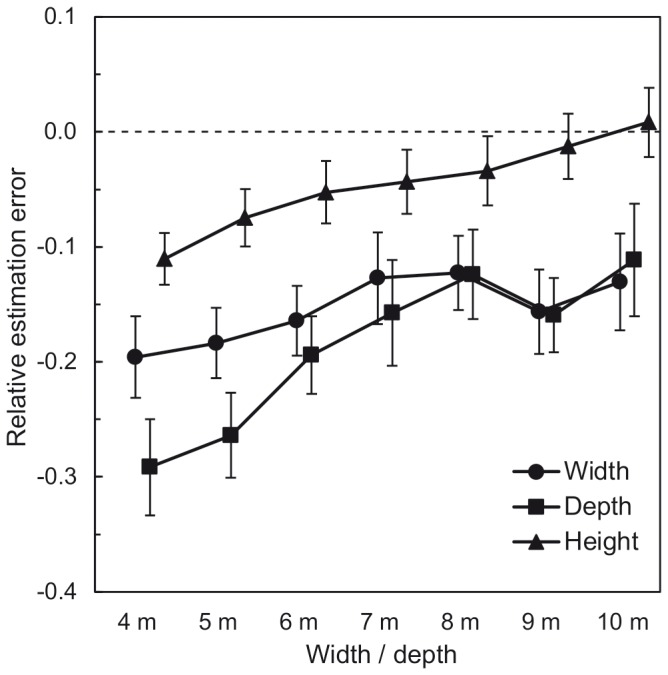
Experiment 2: Mean relative estimation errors of the three room dimensions as a function of width/depth. Error bars show ±1 SEM of the 39 subjects in each condition.

As can be seen in Figure 9, the underestimation of depth and height decreased with increasing surface area. The amount of underestimation of width was less affected by the manipulation of the surface area. The MANOVA showed a significant main effect of surface area. Repeated-measures ANOVAs, which were calculated as a post-hoc analysis, revealed main effects of surface area on depth and height (cf. Table 3). There were no other main or interaction effects (all *p*-values >.05). Trend analyses were conducted for the mean relative estimation errors of height and depth. For height as well as for depth, the decrease of the underestimation with increasing values of width/depth was approximately linear, *F*(1, 228) = 61.365, *p*<.001, *R^2^* = .963 and *F*(1, 228) = 41.247, *p*<.001, *R^2^* = .850, respectively. *R^2^* indicates the proportion of variance explained by the linear model. Both non-linear trend components were not significant, *F*(5, 228) = 0.474, *p* = .795, Δ*R^2^* = .037 and *F*(5, 228) = 1.451, *p* = .207, Δ*R^2^* = .150, respectively. Note that Δ*R^2^* indicates the improvement of the model fit relative to the linear model. Our results are consistent with previous studies that reported an underestimation of depth of interior space in VR, both in verbal estimation tasks (e.g. in meters) and for actions (e.g. throwing a bag or walking blindfoldedly to a target that was seen before) (e.g., [52], [53]). For example, Kunz et al. [52] reported both verbal and action-based (blindfolded walking) underestimation of perceived depth within a range of 3 m to 6 m physical depth, for both low and high quality simulations of classrooms; the mean verbal underestimation was 22.09% and 37.68% for high and low quality simulations, respectively. These values are comparable to the amount of depth underestimation we found in the current study for the two smallest rooms. Underestimation was 29.17% and 26.38% for 4 m and 5 m physical depth, respectively. In contrast, for our largest room (10 m physical depth), the underestimation was only 11.12%. Whereas our data are compatible with a general underestimation of depth of interior space in VR, the decreasing relative estimation error of the depth estimates with increasing physical depth is quite surprising and cannot be explained by general compression effects in VR. According to prior results, the underestimation of depth should have remained constant within the range of 4 m to 10 m. For example, Kunz et al. [52] reported an almost linear relationship between both verbal and action-based depth estimates and the physical depth (3 m to 6 m) of the virtual classrooms. Moreover, for blindfolded walking as well as for timed imagined walking, a linear relationship between perceived and physical magnitude (6 m to 18 m) of exocentric distances was shown by Grechkin et al. [54] both for virtual reality and real life settings. Our findings also differ from previous studies concerned with depth estimates of rectangular enclosed exterior space with depth to width ratios from 10∶1 to 28∶1 and surface areas from 40 m^2^ to 900 m^2^. For such spaces presented on drawings, photographs, and in real life, Gärling [55], [56] reported an increasing underestimation of depth and surface area with increasing physical size. There are several possible reasons for this discrepancy. First, the surface areas of 16 m^2^ and 25 m^2^ for which we found the most distinct underestimation of depth are smaller than the smallest surface area that was used by Gärling. Second, all surface areas in Gärling’s studies had a rectangular shape with depth to width ratios equal to or higher than 10∶1. This indicates that even for the smallest surface area of 40 m^2^, the rear wall was at least 20 m (depth to width ratio 10∶1) away from the observer’s eye. Furthermore, factors like the missing ceiling in outdoor spaces might be a source of inhomogeneous effects in indoor and outdoor spaces. Taken together, we do not have a definite explanation for our result of decreasing underestimation of depth with increasing physical depth and width. Note however, that in outdoor settings, verbal distance estimates also stop to suffer from compression at large distances [57], [58]. For perceived height, the decrease in underestimation with increasing surface area might be due to several factors. Oberfeld and Hecht [1] independently varied the physical height and width of a virtual room and found that physical height affected perceived width, whereas physical width did not affect perceived height. According to these results, an increase in physical width is unlikely to have caused the increase in perceived height. A positive correlation between the room’s physical width and its perceived height would also be incompatible with the architectural rule of thumb that rooms with an increasing width and a constant ceiling height are perceived as increasingly low (c.f. [59]). The influence of volume, surface area, width, and depth on perceived room height appears to be rather complex and deserves further investigation.

The mean relative estimation errors of width, depth, and height suggest a differential compression of the spatial dimensions (see Figure 9). Paired *t*-tests (test value = 0) for the comparisons width vs. depth and height vs. width on the seven levels of surface area were calculated (cf. Table 5). Corresponding to the results of Experiment 1 when the scaling cue was absent, we found a consistent overestimation of height compared to width. As outlined in the results and discussion section of Experiment 1, this finding is consistent with previous research on the perception of horizontal and vertical exocentric distances (e.g., [48], [49]). Furthermore, due to the more pronounced compression of depth relative to width in the two smallest rooms, there was a shift in the perceived shape of the surface area. Thus, the two smallest rooms with surface areas equal to or below 25 m^2^ were perceived as non-square rectangular rooms, whereas rooms with surface areas above 25 m^2^ were perceived as square rooms.

**Table 5:** Experiment 2: Results of the paired-samples t-tests (test value = 0) conducted for the difference of the relative estimation errors in the comparisons width vs. depth and height vs. width, differentiated according to the seven levels of surface area.

Comparison	Surface area	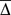	*SE* 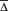	*t*	*df*	*p*	α_corr_
Width vs. depth	16 m^2^	0.096	0.026	3.731	38	<.001*	.007
	25 m^2^	0.080	0.027	2.919	38	.006*	.008
	36 m^2^	0.0300	0.023	1.303	38	.200	.010
	49 m^2^	0.0303	0.024	1.257	38	.216	.013
	64 m ^2^	0.001	0.021	0.055	38	.957	.050
	81 m^2^	0.003	0.026	0.115	38	.909	.025
	100 m^2^	−0.019	0.021	−0.902	38	.373	.017
Height vs. width	16 m^2^	0.085	0.025	3.422	38	.002*	.017
	25 m^2^	0.109	0.026	4.146	38	<.001*	.007
	36 m^2^	0.112	0.030	3.660	38	.001*	.008
	49 m^2^	0.084	0.035	2.389	38	.022*	.050
	64 m ^2^	0.089	0.028	3.146	38	.003*	.025
	81 m^2^	0.144	0.042	3.452	38	.001*	.013
	100 m^2^	0.139	0.039	3.604	38	.001*	.010

*Note:* α*_corr_* indicates the Bonferroni-Holm adjusted alpha level. Significant *p-*values (two-tailed) are marked by an asterisk.

#### Perceived spaciousness

**Figure 10:**
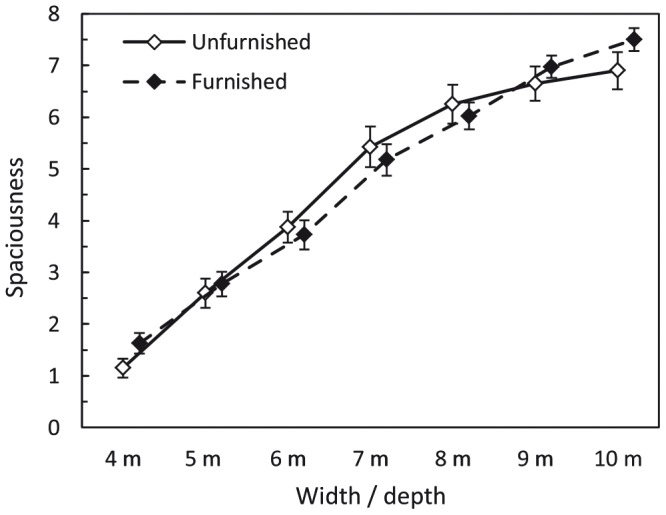
Experiment 2: Mean spaciousness judgments as a function of furnishing and width/depth. Error bars show ±1 SEM of the 40 subjects in each condition.

The mean ratings of spaciousness are displayed in Figure 10. The data were analyzed with a repeated-measures ANOVA using an univariate approach with Huynh and Feldt [60] correction for the degrees of freedom. Furnishing and surface area were within-subjects factors, and perceived spaciousness was the dependent variable. In contrast to Experiment 1, furnishing did not significantly affect the spaciousness judgments, *F*(1, 39) = 0.586, *p* = .449, partial η^2^ = .015. Please see the General Discussion for a possible explanation of this discrepancy. The furnishing×surface area interaction was also not significant, *F*(6, 234) = 1.246, *p* = .285, partial η^2^ = .031, 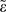 = .963. The effect of surface area was significant, *F*(6, 234) = 149.410, *p*<.001, partial η^2^ = .793, 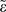 = .987. Consistent with previous results (e.g., [2], [3], [29]), perceived spaciousness increased with increasing surface area. A trend analysis showed that perceived spaciousness increased mainly linearly with increasing width/depth, *F*(1, 234) = 868.707, *p*<.001, *R^2^* = .969. Additionally, we found a small non-linear component which reflects a negatively accelerated increase in spaciousness for values of width/depth above 7 m, *F*(1, 234) = 5.550, *p*<.001, Δ*R^2^* = .031.

## General Discussion

In two experiments, we have investigated the influence of furnishing on the perception of interior space. For the true-to-scale models (Experiment 1), furnished rooms looked less spacious than unfurnished rooms. However, this effect did not translate into the judgments of spatial dimensions. Depth and width ratings were largely unaffected by furniture. Perceived height, in contrast, was increased with furniture. Also, the effect of furnishing to increase perceived height was observed only in the absence of the scaling cue. Thus, our data provide evidence for a complex effect of furnishing on the perception of interior space.

For the room simulations in VR (Experiment 2), furniture had no significant effect on estimates of width, depth and, height, with a tendency towards larger width and depth estimates in the furnished condition. Furthermore, there was virtually no effect of furnishing on perceived spaciousness. Table 6 provides a summary of the reported effects.

**Table 6:** Overview of the effects of furnishing on perceived spatial dimensions and perceived spaciousness of interior space in Experiments 1 and 2.

Experiment	Presentation medium	Effect on	
		Perceived spatial dimensions	Perceived spaciousness
1	Model rooms	Yes	Yes
2	VR	No	No

How do our findings relate to Imamoglu’s studies [18], [21]?

As outlined in the Introduction, Imamoglu reported a negative relation between the degree of furnishing and the room’s perceived volume [18], as well as a reversed U-shaped relation between the degree of furnishing and the room’s perceived spaciousness [18], [21]. In other words: Complete absence of furnishing should maximize the perceived room volume, whereas medium density furnishing should maximize the spaciousness impression.

The increase in perceived height in the furnished condition without scaling cue in Experiment 1 is not compatible with Imamoglu’s [18] finding that the perceived volume of a room decreases with increasing furnishing. Because the increase in perceived height was not accompanied by a decrease in the perceived room width or depth in the furnished condition, the perceived volume should have increased, not decreased. The difference between the studies might be due to the use of different methods. In Imamoglu’s study, observers compared the volume of real size rooms in which they could move about freely, to a real size standard room. Another possible explanation is that the perceived volume of interior space might only be loosely related to the perception of the spatial extension of single dimensions. Furthermore, Imamoglu provided no precise information about of the specifications of the furnishing (e.g. color, style, size).

With respect to spaciousness, our unfurnished model rooms were perceived as larger than the furnished model rooms. At first glance, the direction of this effect contradicts Imamoglu’s [18], [21] finding that furnishing with a medium density increased the room’s perceived spaciousness. However, this apparent contradiction can be explained by differences in the operationalization of spaciousness. Whereas Imamoglu [18], [21], [22] conceptualized spaciousness as a global room impression on the dimensions “appeal”, planning”, and “space freedom”, we regard spaciousness as a more distinct construct, which is defined by a person’s affective appraisal of a room’s narrowness or wideness. Note that the latter operationalization is in accordance with many previous studies on perceived spaciousness (e.g., [2], [3], [4], [26], [27], [28], [29], [36], [37], [38]). In other words, one could argue that medium density furnishing improves the general affective impression of interior spaces, but not the impression of spaciousness in the narrow sense of the word. Interestingly, the latter also seems to be true for Imamoglu’s [21] results: The ratings on the “space freedom”-factor, which he described as “the feeling of roominess as well as the physical size or largeness of the interior” [21] were at a maximum for the empty model room and decreased with increasing furnishing. This is exactly what we found.

Can we conclude then that furnished rooms look higher but less spacious only when observers are confronted with small model rooms?

Before doing so, a few caveats should be considered: First, the effects of furnishing varied with the presentation medium (cf. Table 6). While an effect of furnishing on the perceived spatial dimensions was present when using the true-to-scale model rooms in Experiment 1, no such effect could be found when using the virtual room simulations in Experiment 2. In the latter case, the perceived height remained virtually constant, irrespective of furnishing. However, we found a trend towards larger perceived width and depth of the virtual rooms in the furnished condition. Neither these trends, nor the increase in perceived height in Experiment 1 are compatible with Imamoglu’s [18] data, which were gathered in real life rooms and indicate a decrease in perceived room volume with increasing furnishing density. With regard to a multitrait-multimethod approach [61], the convergent validity of the data so far is poor, and additional research is needed to clarify the influence of different media. Regarding the ratings of spaciousness, the true-to-scale model rooms in Experiment 1 were perceived as less spacious when furnished. For the room simulations in Experiment 2, however, furnishing had no significant effect on spaciousness. Both findings are incompatible with the results by Imamoglu [18], [21]. Note that, as outlined above, the comparability of the results is not ensured due to a very different conceptualization of spaciousness. But how can the discrepancies between the two presentation media in the current study be explained? One possible explanation might be the different availability of depth and size cues. As we found no significant effect of the vision condition (monocular vs. binocular) in Experiment 1, a lack of stereoscopic cues is unlikely to have caused the furniture effects to vanish in the virtual reality experiment. Motion-induced cues were comparable in both experiments, as participants could slightly move their head while rating the model rooms, as well as change their virtual line of sight by turning the mouse wheel while rating the virtual rooms. Regarding monocular cues, the major difference lay in the virtual rooms’ more pronounced room boundaries in the virtual reality experiment compared to the model room experiment. The fine-grained texture of the surfaces and the shadowing of the edges and corners in the virtual reality experiment might have overemphasized the rooms’ boundaries. As our low-density furnishing stimuli revealed relatively more of the surfaces, edges, and corners, we surmise that the increased salience of the room boundaries has nulled the effects of furnishing in the virtual reality experiment.

A second aspect relates to the density and arrangement of furnishing. With a maximum percentage of 11.84% of surface area covered by furniture, we used low furnishing densities. In contrast, the maximum furnishing density realized by Imamoglu [18] was approximately 41%. Besides the density, the arrangement of furnishing (e.g. centered vs. near-wall, spread out in the room vs. crowded together) is also likely to influence the perceived spatial dimensions as well as the impression of spaciousness. The common rules of thumb in architecture and interior design emphasize the importance of both the role of furnishing density and the role of arrangement of furnishing. For example, a near-wall furnishing is said to optically enlarge interior space [12]. More sophisticated rules regarding the arrangement of furnishing might be derived from Gestalt psychology (e.g., [62], [63]). For example, the rule of proximity, according to which two or more objects being close to each other appear as belonging together, might also affect the perceived extension of the wall behind these objects.

Third, providing subjects with a cue of familiar size in Experiment 1 suppressed effects of furnishing on perceived room height. To a lesser degree, this effect can also be expected for other objects with familiar size, such as doors or windows. Such cues are ubiquitous in real life rooms, and experimental approaches with true-to-scale model rooms or three-dimensional simulations in VR have the advantage that the absence/presence of these cues can be controlled. Besides these methodological considerations, this finding also raises the question of the relative potency of different cues for the spatial extent of interior spaces. To provide a concrete example for this issue: What would the perceived spatial extent of an interior space be, say a model living room, with oversize furnishing but normal size puppets or avatars compared to the same interior space with normal size furnishing but oversize avatars?

Fourth, characteristics of the furnishing, such as shape, color, or the level of abstraction (e.g. abstract cuboids vs. real life furniture) might moderate potential effects of furnishing on the perceived size of interior space. With this in mind, we used very abstract and simplified objects to test their influence on perceived spatial dimensions and spaciousness. This is a limitation of the present study. Moreover, even characteristics of the surrounding room, such as surface area shape (e.g. square, rectangular, or elliptical), provide many additional variation possibilities. In this context, the interplay between characteristics of the furnishing and the surrounding room should be of special interest – for example, the independent variation of furnishing height and room height.

Given our use of model rooms and virtually rendered interior spaces, there might be restrictions in the external validity of the results to be considered. A number of studies (e.g., [2], [3], [4], [26], [27], [28], [29], [36], [37], [38]) have demonstrated the impact of design characteristics of virtual interior spaces on spaciousness ratings (in the sense of internal validity). However, next to nothing is known about the transferability of spaciousness ratings from virtual reality experiments to spaciousness ratings in genuine reality. We are not aware of any study that compares spaciousness ratings gathered in virtual rooms with ratings gathered in real life interior spaces. However, with reference to Imamoglu, who compared spaciousness ratings from 1∶10 model conference rooms [21] with ratings gathered in real life office rooms [18], we assume the spaciousness ratings from model rooms to be transferable to real life rooms. In terms of the perceived spatial extent of room dimensions, Kunz et al. [52] and Grechkin et al. [54] reported an almost linear relationship between both verbal and action-based depth estimates and the physical depth. Note that the latter two studies found a general underestimation of distances in virtual reality and that this finding is not ad odds with assumption of comparable effects in virtual and genuine reality. When interested in the effect of manipulations of interior space design characteristics on the perceived spatial extent of room dimensions relative to a baseline measurement, the absolute level of the estimates is irrelevant as long as the direction and the slope of the effects remain unaffected. Regarding model rooms, we are not aware of any study to compare the perceived spatial extent of single room dimensions of model rooms with that of real life rooms. However, with respect to the perceived volume of differently shaped rectangular rooms, prior studies. [31], [32], [64] reported comparable results for model and full-size rooms.

Potential reliability restrictions of the verbal room dimension estimates are also worth discussing. Kunz et al. [52] compared the variable (intra-individual variances of three repeated measurements of the same depths) errors for verbal and action-based depth estimates. The mean variable errors were very similar for the two shorter depths. However, for 6 m physical depth, there was a slight increase in the variable error of the verbal estimates compared to the action task. Taken together, we take verbal estimates of single room dimensions to be sufficiently reliable.

Which conclusions can be drawn from the present study?

There are three major implications of our data: First, perceived spatial dimensions and spaciousness are not the same. The data from Experiment 1 indicate that the perception of the spatial dimensions and spaciousness of interior space do not inevitably follow the same rules. Note that this is compatible with Imamoglu’s [18] findings. Second, there is an effect of furnishing on the perceived spatial dimensions of interior space which is worthwhile to study in greater detail. Third, there is an effect of furnishing on the perceived spaciousness of interior space. For the latter, our results suggest that the relation between furnishing and perceived spaciousness cannot always be assumed to be a reversed U-shaped function as proposed by Imamoglu [18], [21].

The current study has shown that the relationship between the perception of interior space and furnishing is much more complex than thought previously. For example, it is by no means obvious that a realtor should present a furnished apartment as opposed to an unfurnished in order to make it look maximally spacious.

## Supporting Information

File S1 **Raw data** **of Experiment 1 and Experiment 2.** Archive file containing the datasets of Experiment 1 (true-to-scale model rooms) and Experiment 2 (virtual rooms).
(ZIP)
